# Out-of-clinic patient communication in paediatric rheumatology: the extent and nature of demand

**DOI:** 10.1186/1546-0096-11-13

**Published:** 2013-03-27

**Authors:** Debi V Feldman, Jo Buckle, Jane E Munro, Roger C Allen, Jonathan D Akikusa

**Affiliations:** 1 Rheumatology Service, Department of General Medicine, The Royal Children’s Hospital, Flemington Rd, Parkville, VIC 3052, Australia; 2 The Murdoch Childrens Research Institute, Melbourne, Australia

**Keywords:** Juvenile idiopathic arthritis, Paediatric rheumatology, Communication, Patient-centered care

## Abstract

**Background:**

Traditional funding models for public paediatric rheumatology care are typically based on providing medical services for a defined number of clinics per week. Anecdotally there is significant demand by patients and families for out-of-clinic communication with care providers and services provided under traditional funding models may not meet this need. Our aim was to determine the extent and nature of this ‘hidden’ demand in a tertiary paediatric rheumatology centre.

**Methods:**

Communication data and diagnoses were extracted from the Rheumatology service database at our centre for the period 1/1/2009 to 31/12/2011. Clinical activity data over the same time were obtained from hospital clinic databases.

**Results:**

There were 5672 instances of communication with 749 patients/families over 3 years, (mean 7.3/weekday). This increased over time in parallel with clinical activity. 41% of clinic patients sought communication with the team out of clinic hours. 58% were telephone calls, 36% emails and 6% letters. The communication topics were for advice, results or general updates (28%), medication queries (24%), appointment/admission coordination (20%), disease flare or other disease events (14%), psychosocial, school or transition issues (6%) and miscellaneous queries (8%). Of the most frequent communicators, those with juvenile idiopathic arthritis were the majority (85%). The remainder had other chronic inflammatory conditions.

**Conclusions:**

The communication and support needs of patients with chronic rheumatic diseases and their families extend beyond that which can be provided in the clinic environment. It is essential that funding for paediatric rheumatology services allows for staffing sufficient to meet this need.

## Background

Health systems are often designed to manage acute illness [1]. Modern health care models recognize that this traditional acute focus often does not adequately meet the needs of people with chronic illness. These newer models emphasize the importance of a patient-centered focus, as recently described by the American Academy of Pediatrics [2]. They empower patients and families to develop self-management skills and recognize that even with these skills patients and families may need to interact with health care providers outside standard clinic hours [1,3]. Peak bodies such as the British Society of Paediatric and Adolescent Rheumatology advocate for a holistic approach, which includes provision of a dedicated telephone helpline as part of the minimum standards of care for young people with juvenile idiopathic arthritis (JIA) [4,5]. Despite these recommendations, little has been published about the nature and level of demand for this type of service in patients with paediatric rheumatic diseases and their families.

Paediatric rheumatology services provide care for children and young people with chronic inflammatory and non-inflammatory conditions, of which the most common is JIA. Most of this care is provided in an outpatient setting. In Australia, funding of public paediatric rheumatology services is based on provision of medical and allied health care for a defined number of clinics per week. Despite global trends in models of care for chronic illness, there is no dedicated funding for the provision of support to patients and families outside of the clinic setting. The rheumatology service at the Royal Children’s Hospital (RCH) in Melbourne, is the larger of two services based in tertiary referral centres providing care to a population of 5.6 million people in the State of Victoria, Australia. The service comprises three paediatric rheumatologists, and one rheumatology nurse and one service coordinator, both of whom are philanthropically funded. The nurse and service co-ordinator are the designated primary point of out-of-clinic contact for patients and families. Each contact is documented in the service’s computerized clinical database. Anecdotally, in our patient group, there is significant demand by patients and their families for this support. The aim of this study was to examine database entries documenting all out-of-clinic communications between patients and their families and the rheumatology nurse and co-ordinator over a 36 month period to determine the nature and extent of demand for this support.

## Methods

A review was conducted of all entries in the computerized clinical database maintained by the rheumatology service related to patient or family communications with the paediatric rheumatology nurse or co-ordinator between 1/1/2009 to 31/12/2011. The database is a comprehensive clinical tool coded in Microsoft Access™ in which patient demographic details, diagnoses, medications, clinic visits, internal patient-related team communications and external communications with patients, their families and community care providers, are recorded prospectively on searchable proformas. For communications, database entries include the date, type (e.g. telephone call, email, letters), staff member involved, and allow free text for a description of the specific content of the communication. Compliance rate with data entry is high as the contents are the only record of patient-related activity kept by the service and printed outputs are sent to the patients’ hospital file.

Data relating to demographics, communication content, and clinic attendance during the period of interest were abstracted to a Microsoft Excel Spreadsheet. Reasons for communication were examined by taking a sample of data for one month (March) in each year over the period of interest. The free text content recorded for each communication was assigned to one of six broad topic categories by author consensus.

Trends in out-of-clinic communication over time were examined by plotting the number of communication events by month over the period of interest. To determine how these trends compared with the overall clinical activity of the service, the number of new and review patients seen in clinics, obtained from attendance lists maintained by the RCH outpatients department, were similarly plotted. To determine what proportion of patients seen in clinics communicate with the nurse or coordinator, the clinic lists of two clinicians who work exclusively with public patients were reviewed, and the number of patients from these lists who had communicated with the service out of clinic hours was noted.

To examine whether communication with the service was more likely to occur for patients with a given diagnosis, patients and families were ranked by frequency of communication with the service, and the diagnoses of the top 10% of communicators by frequency were then examined.

Descriptive statistics were used to describe all data. This study was approved by the RCH Research Ethics Committee.

## Results

**Figure 1:**
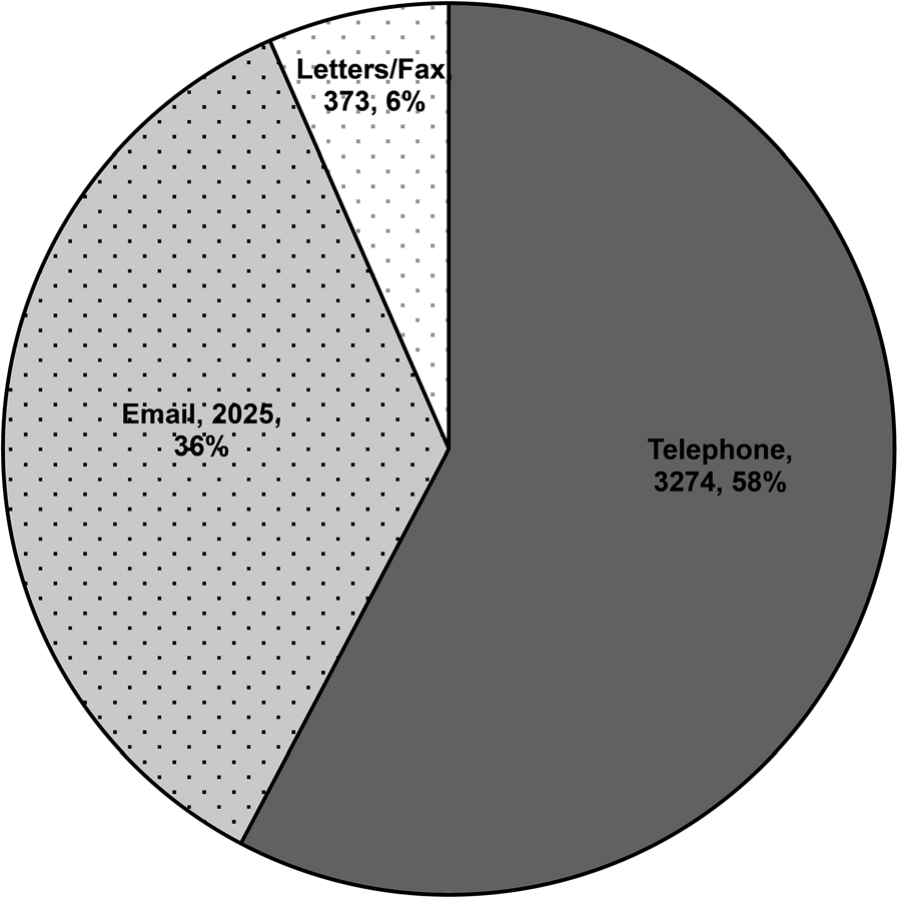
**Preferred means of out-of-clinic communication between the paediatric rheumatology nurse or co-ordinator and patients/families.** Numbers are total instances of that modality noted. Percents are proportions of the total communications received.

Between 1 January 2009 and 31 December 2011 there were 5672 instances of out-of-clinic communication (mean: 7.3/weekday) with the paediatric rheumatology nurse or co-ordinator, involving 749 individual patients or their families. The preferred means of out-of-clinic communication are shown in Figure 1.

**Figure 2:**
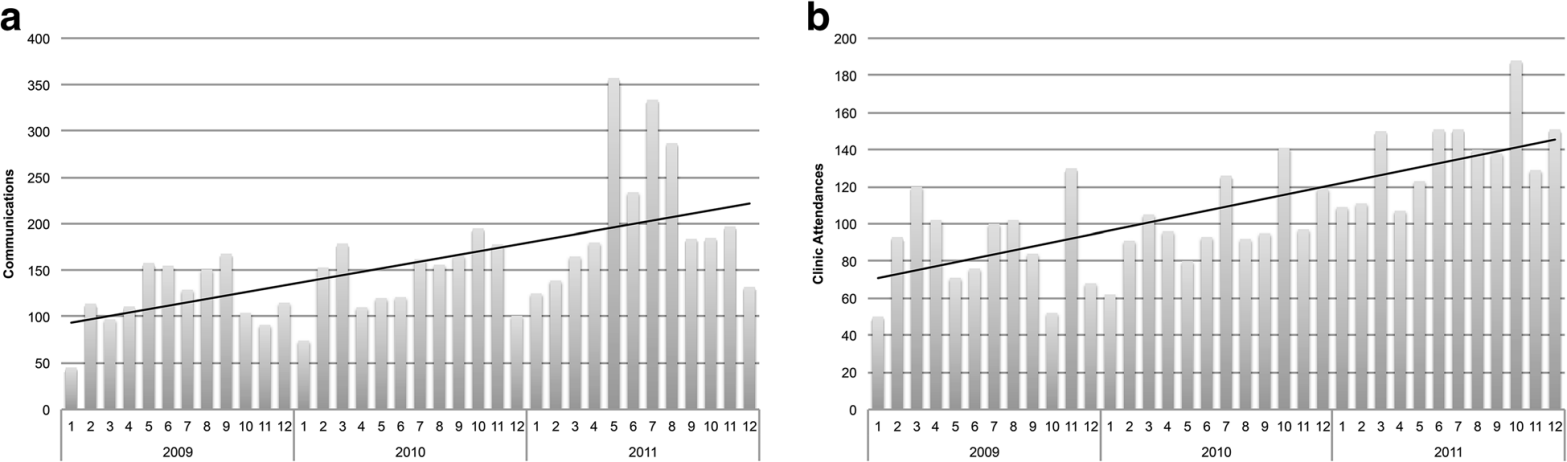
**a: Number of out-of-clinic communications between the paediatric rheumatology nurse or co-ordinator and patients/families.** Number of instances of communication each month from 1/1/2009 to 31/12/2011. **b***:* Clinical activity: patient attendance at a paediatric rheumatology clinic. Number of attendances each month from 1/1/2009 to 31/12/2011.

Reasons for communication with the rheumatology nurse or co-ordinator were examined for 439 contact episodes, as shown in Table 1. Requests for advice included requests for allied health plans and information about JIA. Disease flares or other disease events included complaints of joint pain or pain following injection. Miscellaneous issues included requests for sick certificates, visa paperwork or letters of support. Over the study period there was a trend of increased out-of-clinic communication over time which paralleled an increase in the service’s clinic activity (Figure 2a and b).

**Table 1:** Reasons for communication for a sample of 439 contact episodes

	**Number (Percent)**
Request for advice, results or general update	122 (28)
Medication query	107 (24)
Appointment or admission coordination	86 (20)
Flare or other disease event	62 (14)
Psychosocial, school, transition issue	25 (6)
Miscellaneous	37 (8)
**Total**	**439 (100)**

Examination of the clinic statistics for the two clinicians who only see public patients revealed they had seen 1082 individual patients on 3583 separate occasions over the study period. Of these, 444 (41%) made out-of-clinic contact with the rheumatology nurse or co-ordinator on 3697 separate occasions. For the patients or families who communicated with the service outside-of-clinic, the number of contacts per patient/family over the study period ranged from 1–144 (median 3 times).

Patients or families in the top 10% of communicators with the team by frequency of contact made up the majority (55%, or 3092 instances) of the total out-of-clinic communications dealt with by the service. These patients or families communicated with the team between 21 and 144 times (median 34 times) over the three years. Of these most frequent communicators, those with juvenile idiopathic arthritis made up the majority (total of 85%; polyarticular 36%, oligo or oligo-extended 21%, enthesitis or undifferentiated 15%, systemic 13%). The remainder had other chronic inflammatory conditions with diagnoses of systemic lupus erythematosus (7%), mixed connective tissue disease (4%), familial Mediterranean fever (3%) and chronic recurrent multifocal osteomyelitis (1%).

## Discussion

Information about the extent and nature of demand for support from health care providers outside the clinic setting is important for informing the development of public health services that meet the needs of patients with chronic illness such as JIA. This study is the first to look at the demand for out-of-clinic support in patients with paediatric rheumatic diseases using data prospectively collected over a prolonged period. We found that there was substantial demand by patients and families for communication with our paediatric rheumatology nurse and co-ordinator out of clinic hours, with an average of 7.3 instances of communication per weekday. This is comparable with use of a telephone helpline for a tertiary referral paediatric rheumatology service in the United Kingdom, where an average of 5.5 calls were received per working day over a one month period, but less than the median of 21 incoming calls per day reported at a paediatric neurology unit in Canada [6,7].

We found that those most frequently seeking support were parents of patients with chronic inflammatory rheumatic diseases, particularly juvenile idiopathic arthritis. Examination of the reasons for communication revealed an important range of concerns, with more than half of communications related to requests for advice about disease management, results, disease status updates, or queries about medication. If left unaddressed until the patients’ next clinic visit, these issues could potentially result in adverse clinical outcomes.

Over the period examined there was a year-on-year increase in out-of-clinic communication with patients and families, which paralleled an increase in the number of patients seen in clinic by our service. The finding that 41% of patients seen in clinic seek support from the service outside of the clinic environment would explain this parallel increase and suggests that an increase in the clinical service provision of a paediatric rheumatology unit must also include a capacity to manage an increase in demand for out-of-clinic support.

Little has been published regarding the demand for out-of-clinic communication with health care providers in paediatric patients with chronic diseases and their families. This despite the fact that access to out-of-clinic communication with the treating clinician has been found to improve parental perceptions of quality of care in the paediatric rheumatology population [8]. Those that have been published show significant demand for telephone support from service providers in the setting of a paediatric neurology service [7] and from parents of children with congenital anomalies following discharge from hospital [9]. There is more data in adult chronic disease populations where a significant demand for telephone support has been shown [10-12]. Providing telephone support is becoming an integral part of adult rheumatology care in England and Wales [13]. It is valued by patients and enhances clinical service provision [14].

Lack of recognition of the demand for, and funding to provide, out-of-clinic communication and support, may act as a barrier to the optimal management of children and young people with chronic rheumatic illness. In addition to improving the quality of care, addressing this need may also have cost advantages in reducing the requirement for clinic reviews and unplanned attendances in emergency departments. For adults with chronic obstructive pulmonary disease, access to telephone support has been associated with a reduction in hospital admission [11,12], and for one adult rheumatology unit, 60% of patients surveyed indicated that they would have attended a healthcare provider had the telephone support not been available [15]. The extent of out-of-clinic communications also reveals a potential unmet demand by patients and families for further education and support. Future research regarding this issue may enhance service provision by informing the development of new models of patient care and support.

This study has several limitations that should be considered when interpreting its results. We did not capture out-of-clinic communications between patients/families and medical staff or members of the multi-disciplinary team other than the nurse and unit co-ordinator, such as social worker, physiotherapist or occupational therapist. The results therefore underestimate the communications with the entire team over the study period. The magnitude of this underestimate, however, is likely to be small as the nurse and co-ordinator are the nominated primary out-of-clinic point of contact with the service for patients and their families. The examination of the reasons for communication with the team included only a sample of all communications recorded. As such there is the possibility that the proportion of communications in each category in the time periods sampled may differ from those across the whole dataset. The month chosen (March) is a ‘typical’ clinical month with no school/university holidays and staff leave rare (just after the long summer break in Australia), therefore we feel any differences are likely to be small. The study did not capture the time spent dealing with patient communications, making it difficult to assess the workload these communications generate. In the study by Fountain-Polley et al. it was estimated that the 5.5 calls per day to their paediatric rheumatology helpline created, on average, 54.4 minutes of work per working day [6]. As telephone contact was the most common mode of communication with our patient group, the effect on the workload for our service may be similar.

This study also has some important strengths. The database used is the sole means of recording patient interactions for the nurse and co-ordinator, which occurs as standard operating procedure. Therefore, information regarding their communication with patients and families is likely to be accurate and complete. Use of the database as standard practice within the service has also allowed us to examine communications over a prolonged period, providing an accurate assessment of the need for this form of support over time.

## Conclusions

In this study we have shown that the communication and support needs of patients and families with paediatric rheumatic illnesses extend beyond that which can be provided in the clinic environment. This out-of-clinic demand for support increases in parallel to the clinical load of the service. Funding models based on the provision of a set number of clinics per week will be inadequate to service this need. In the context of modern models of care for patients with chronic disease, which emphasize a patient-focused approach and place value on enhancing communication between patients and care providers, it is essential that funding for paediatric rheumatology services allow for staffing sufficient to meet this need.

## Competing interests

The authors declare that they have no competing interests.

## Authors’ contributions

All authors participated in study concept. DF, JB and JA designed and conducted the study. DF wrote the manuscript and all authors participated in drafting and approved the manuscript to the final version.
